# Recent Developments in Direct C–H Functionalization of Quinoxalin-2(1*H*)-Ones via Heterogeneous Catalysis Reactions

**DOI:** 10.3390/molecules28135030

**Published:** 2023-06-27

**Authors:** Qiming Yang, Hu Wang, Xiang Wang, Yizhu Lei

**Affiliations:** Guizhou Provincial Key Laboratory of Coal Clean Utilization, School of Chemistry and Materials Engineering, Liupanshui Normal University, Liupanshui 553004, China; wanghu301@163.com (H.W.); 15348524984@163.com (X.W.)

**Keywords:** C–H functionalization, heterogeneous catalysis, heterocyclic compounds, quinoxalin-2(1*H*)-ones

## Abstract

In recent years, Web of Science has published nearly one hundred reports per year on quinoxalin-2(1*H*)-ones, which have attracted great interest due to their wide applications in pharmaceutical and materials fields, especially in recyclable heterogeneous catalytic reactions for direct C–H functionalisation. This review summarises for the first time the methods and reaction mechanisms of heterogeneous catalytic reactions of quinoxalin-2(1*H*)-ones, including six major types of heterogeneous catalysts involved. The heterogeneous reactions of quinoxalin-2(1*H*)-ones are summarised by classifying different types of catalytic materials (graphitic phase carbon nitride, MOF, COF, ion exchange resin, piezoelectric materials, and microsphere catalysis). In addition, this review discusses the future development of heterogeneous catalytic reactions of quinoxalin-2(1*H*)-ones, including the construction of C-B/Si/P/R_F_/X/Se bonds by heterogeneous catalytic reactions, the enrichment of heterogeneous catalysts such as metal oxides, graphene-based composites, doped metal nanoparticles, and molecular sieve-based porous materials, asymmetric synthesis, and other areas. The aim of this review is to contribute to the development of green and sustainable heterogeneous reaction methods for quinoxalin-2(1*H*)-ones with applications in materials chemistry and pharmacology.

## 1. Introduction

**Scheme 1:**
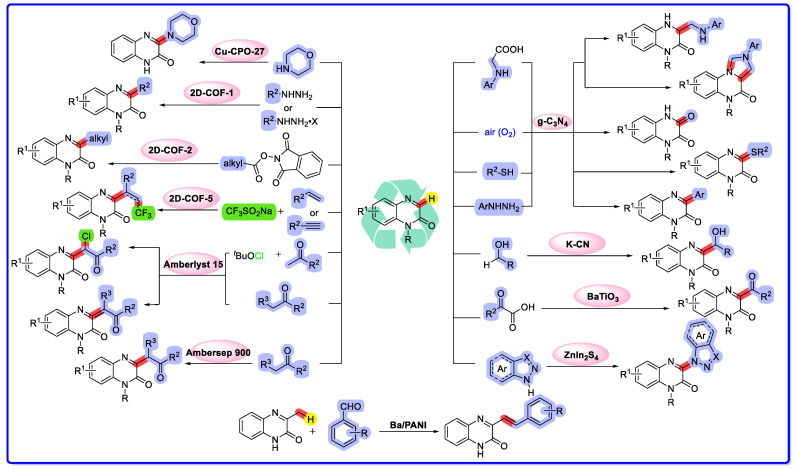
Direct C–H functionalization of quinoxalin-2(1*H*)-ones via heterogeneous catalysis reactions.

Catalysis is of vital importance in both fundamental research and industrial applications. At the heart of catalytic science, heterogeneous catalysis has gained widespread attention for its low cost, environmental friendliness, high activity, selectivity, and stability, making catalysts easy to recycle, regenerate, and sustainably produce [1]. As a result, it plays an integral role in the production of over 80% of the world’s chemical products [2]. In the development of high performance heterogeneous catalytic reactions, a wide range of heterogeneous catalysts have been developed, including solid acid catalysts, organic base catalysts, metal catalysts, metal oxide catalysts, complex catalysts, rare earth catalysts, molecular sieve catalysts, biocatalysts, nanocatalysts, single-atom catalysts [3], and more. Ye and Wu et al. [4] recently used microalgal carbon-based magnetic solid acid catalysts to catalyse the production of biodiesel from wet microalgae. Fu et al. [5] used solid acid catalysts integrated with ceramic membranes to facilitate amine-based CO_2_ desorption. Romero-Zerón et al. [6] reported the preparation of organic base catalysts for biodiesel from animal shells and metal oxides. Heterogeneous catalysts of metals and metal oxides on ordered mesoporous carbon and metal oxide-supported metal catalysts for electrocatalytic oxygen reduction reactions have recently been reported in reviews [7,8]. Progress in research on metal oxide-based biodiesel nanocatalysts has been summarised by Kumar’s group [9]. Bao and his colleagues summarised the metal oxides used for the electrocatalytic reduction of carbon dioxide [10]. Rehman and his colleagues reported the design of a hybrid g-C_3_N_4_/ZnO-W/Co_x_ heterojunction photocatalyst for the removal of methylene blue dye [11]. Mono- and multi-nuclear pincer-type Ru(II) complex catalysts and their catalytic applications have been summarised by Yu’s group [12]. Bifunctional hybrid rare earth solid catalysts for the production of biodiesel from acidic palm oil were reported in [13,14], and the application of rare earth-based nanomaterials in electrocatalysis was reviewed. Liu and Song’s research group summarised the research progress on metal molecular sieve catalysts for propane dehydrogenation [15]. The role and application of biocatalysts in cancer drug discovery has recently been reviewed as well [16]. Li and colleagues recently summarised targeted intermetallic nanocatalysts for sustainable biomass and CO_2_ value addition [17]. Javed and colleagues reported salt-assisted gas–liquid interfacial fluorine doping of metal-free electrocatalysts [18]. Decades of research into these materials have produced many catalysts of great scientific and industrial importance. Heterogeneous catalysis is crucial for the development of organic synthetic methods, especially for the study of multiphase catalysis of parent core structures such as quinoxalin-2(1*H*)-one, which possess a wide range of biological activities and functionalities. In recent years, heterogeneous catalytic reactions using quinoxalin-2(1*H*)-one as a substrate have received increasing attention (Scheme 1). A number of ingenious and innovative methods have been developed, showing great promise for this promising compound with many applications.

**Scheme 2:**
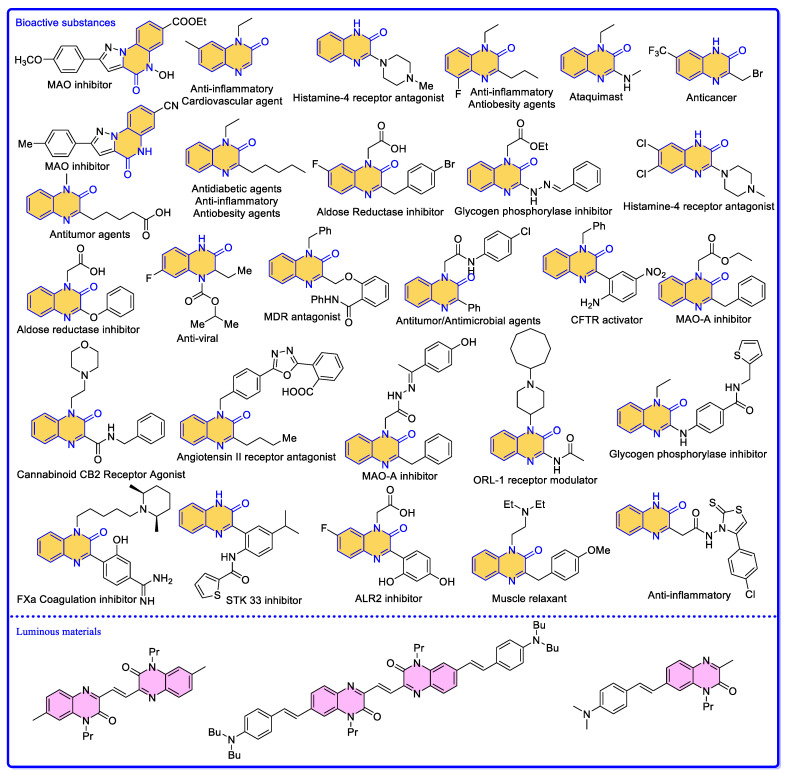
Bioactive substances and luminous materials related to quinoxalin-2(1*H*)-one compounds.

Quinoxalin-2(1*H*)-one is considered to be an important class of nitrogen-containing heterocyclic structural groups found in a variety of biologically active natural products and pharmaceutical compounds (Scheme 2) [19,20,21]. Quinoxalin-2(1*H*)-ones have a wide range of applications in materials science as well (Scheme 2) [22,23]. The direct C–H functionalisation of quinoxalin-2(1*H*)-one at the C3 position is the most cost-effective method for the synthesis of quinoxalin-2(1*H*)-one derivatives, and a large number of studies have appeared in this field in recent years [24,25,26,27,28,29,30,31,32,33,34,35]. A Web of Science search revealed that the number of papers related to quinoxalin-2(1*H*)-one has increased year by year over the last five years, with a total of 25 in 2018, 57 in 2019, 61 in 2020, 92 in 2021, and 95 in 2022. Thus far, there have been 39 relevant articles published in 2023. In particular, studies on the heterogeneous catalytic reactions of quinoxalin-2(1*H*)-ones are emerging due to their environmental friendliness, high activity, selectivity, stability, ease of recovery, regeneration, and sustainable production. Despite these achievements, C–H functionalisation studies of quinoxalin-2(1*H*)-one using heterogeneous catalysts have not yet been reported in a review. A summary of the results of quinoxalin-2(1*H*)-one in the field of heterogeneous catalytic reaction studies is essential for the development of quinoxalin-2(1*H*)-one in the field of heterogeneous catalytic reaction studies. Therefore, it is necessary to review them in order to better develop C–H functionalization methods for quinoxalin-2(1*H*)-one using heterogeneous catalysts. In this paper, for the first time, we review the recent research progress on heterogeneous catalytic reactions for the C–H functionalisation of quinoxalin-2(1*H*)-one and discuss the corresponding reaction mechanisms. We have classified the heterogeneous catalysts according to their types.

## 2. Direct C–H Functionalization of Quinoxalin-2(1*H*)-Ones via Heterogeneous Catalysis

### 2.1. Graphitic Phase Carbon Nitride as a Heterogeneous Catalyst

Graphite carbon nitride (g-C_3_N_4_) is a low cost, easy to prepare, and metal-free semiconductor photocatalyst. g-C_3_N_4_ has emerged as a powerful method for various organic transformations as an environmentally friendly, easily recyclable, and efficient heterogeneous photocatalyst [36,37,38,39,40].

**Scheme 3:**
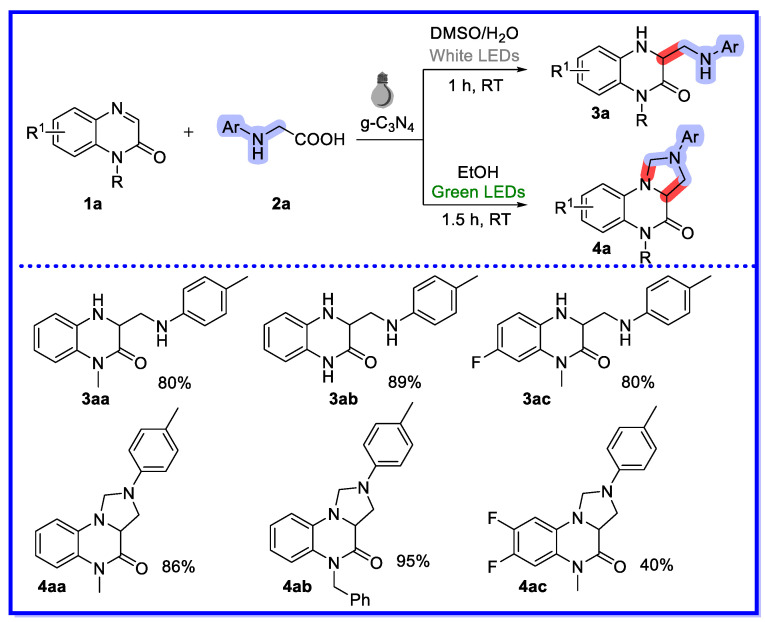
g-C_3_N_4_-catalyzed divergent heterogeneous reaction.

In 2020, Yu and colleagues reported the g-C_3_N_4_-catalyzed divergent heterogeneous reaction of quinoxalin-2(1*H*)-one with N-arylglycine under visible light (Scheme 3) [41]. The solvent-controlled reaction resulted in good yields of the products dihydroquinoxalin-2(1*H*)-ones and tetrahydroimidazo [1,5-a]quinoxalin-4(5*H*)-ones when the reactions were carried out in DMSO/H_2_O and EtOH, respectively. A series of substrates were completed and good yields were obtained. The product dihydroquinoxalin-2(1*H*)-ones was reacted in 80% yield at the gram level. After a series of controlled experiments, it is reasonable to conclude that the reaction medium is the key modulator of this switchable conversion, that is, low concentrations of oxygen in DMSO/H_2_O produce the hydrogen ammonia methylation product **3a**, whereas the cyclization product **4a** is favoured when the reaction is carried out in ethanol with high dissolved oxygen concentrations. Recirculation experiments of g-C_3_N_4_ were investigated, and the catalytic activity of g-C_3_N_4_ remained essentially unchanged in the fifth run after ethyl acetate washing, indicating that this reusable multiphase photocatalyst is stable and reproducible.

**Scheme 4:**
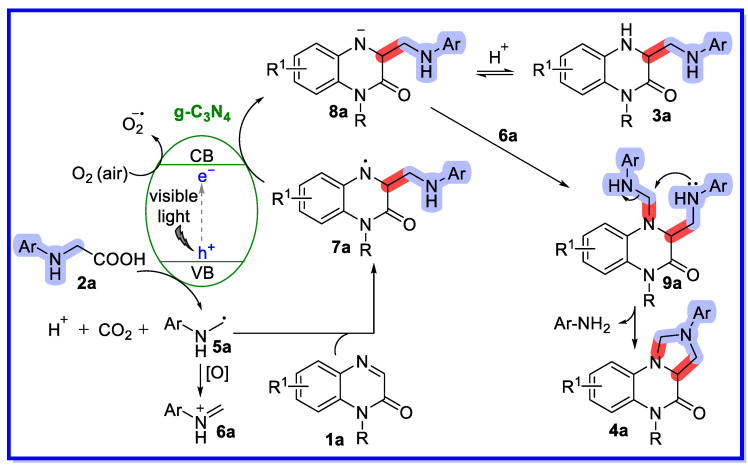
Reaction mechanism catalyzed by g-C_3_N_4_.

A free radical reaction mechanism has been proposed, as shown in Scheme 4 [41]. Upon irradiation with visible light, the semiconductor g-C_3_N_4_ was excited to generate electrons and holes, which play an important role in organic transformations by acting as single electron acceptors and donors, respectively. First, the radical intermediate **5a** was formed through a photo-oxidation process by releasing a CO_2_ molecule and a proton. The radical **5a** was then added to the C=N bond of **1a** to provide intermediate **7a**. Intermediate **7a** then underwent a single electron photoreduction to produce the anion **8a**. The intermediate anion **8a** could be converted into the product **3a** after protonation. On the other hand, the oxidation of the radical **5a** provided the iminium species **6a**. The combination of the iminium species **6a** and the anion **8a** provided the intermediate **9a**, followed by intramolecular cyclization to provide the product **4a** through the release of ArNH_2_. The presence of aromatic amines was confirmed by electrospray ionisation (ESI) mass spectrometry.

**Scheme 5:**
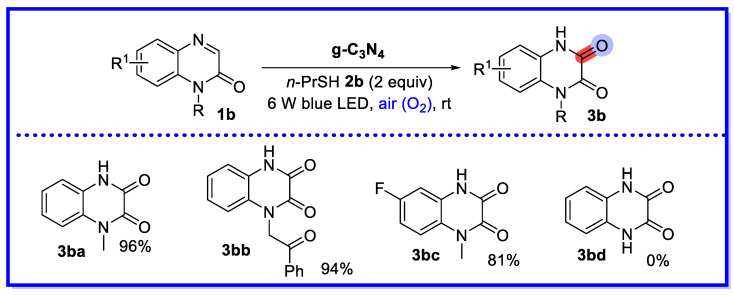
Heterogeneous hydroxylation of quinoxalin-2(1*H*)-one catalyzed by g-C_3_N_4_.

In 2022, Peng and co-workers proposed a heterogeneous visible light-induced direct C–H hydroxylation of quinoxalin-2(1*H*)-ones (Scheme 5) [42]. The method uses g-C_3_N_4_ as a photocatalyst under air conditions and yields a variety of 3-hydroxyquinoxalin-2(1*H*)-ones by simple washing and filtration. The hydroxylation reaction of quinoxalin-2(1*H*)-one was studied cyclically under standard conditions. At the end of the reaction, the g-C_3_N_4_ photocatalyst was recovered and dried from the reaction mixture by simple filtration and rinsing with the reaction solvent, then directly reused in the next round. The reaction was repeated up to six times with no significant loss of catalytic activity. It is worth noting that the reaction does not proceed smoothly in the absence of thiols or in an N_2_ environment. This indicates that thiols and oxygen are necessary for the reaction. An identical reaction was carried out in a homogeneous catalytic system using a DL-α-tocopherol methoxy-polyethylene glycol succinate solution (2 wt% water) (TPGS-750-M/H_2_O) as catalyst. However, this approach has the disadvantage of using large excesses of environmentally unfriendly inorganic strong oxidants and harsh reaction conditions, which generates chemical waste and increases manufacturing costs [43].

**Scheme 6:**
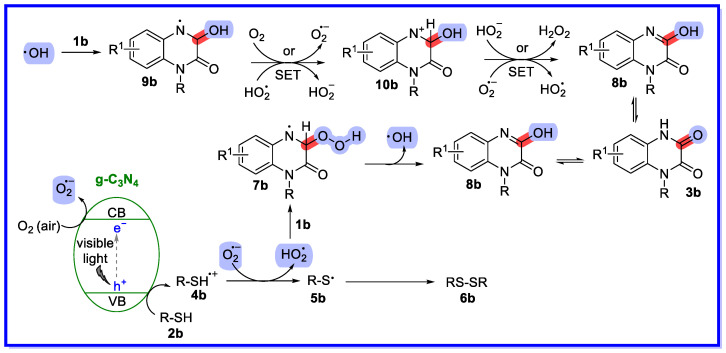
Reaction mechanism of hydroxylated quinoxalin-2(1*H*)-one.

Based on controlled experiments and references in the literature, the reaction mechanism shown in Scheme 6 was proposed [42]. First, g-C_3_N_4_ is excited by visible light to produce an electron in the conduction band (CB) and a hole in the valence band (VB). The VB hole then gains an electron from the thiol **2b** to form the thiyl radical cation **4b** through a single electron transfer process. At the same time, an electron from the CB is transferred to O_2_ (air) to form O_2_^•−^, which has been confirmed by EPR analysis using 5,5-dimethyl-1-pyrroline (DMPO) as a spin trapping agent. Next, O_2_^•−^ abstracts hydrogen from the thiyl radical cation **4b** to form HO_2_• species and thiyl radical **5b**. The resulting HO_2_• can then selectively attack the C-3 position of **1a** to form intermediate **7b**. Intermediate **7b** may undergo homolytic cleavage under visible light irradiation to produce **8b** and the hydroxyl radical HO•. The hydroxyl radical HO• adds to the C=N of **1b** to provide the nitrogen radical intermediate **9b**, which undergoes further oxidation by HO_2_• or O_2_ to provide the nitrogen cation intermediate **10b**. Deprotonation of intermediate **10b** provides intermediate **8b**. Finally, intermediate **8b** undergoes rapid tautomerisation to provide the more stable product **3b**. Meanwhile, the thiyl radical **5b** can rapidly dimerise to form disulfides **6b**.

**Scheme 7:**
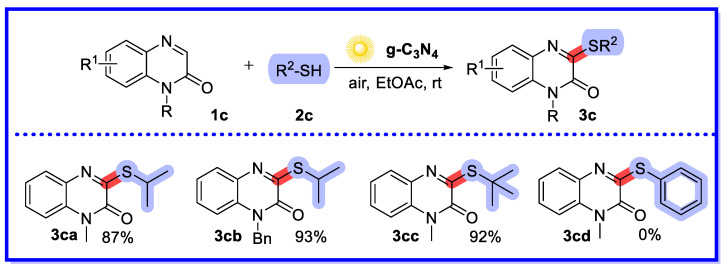
g-C_3_N_4_-catalysed sulfenylation reaction of quinoxalin-2(1*H*)-ones.

In the same year, Xie et al. reported a sunlight-induced and g-C_3_N_4_-catalysed sulfenylation reaction of quinoxalin-2(1*H*)-ones under air conditions (Scheme 7) [44]. The reactions of this method offer a very attractive and practical way to selectively obtain a wide variety of 3-sulphated quinolin-2(1*H*)-ones in good to excellent yields. In addition, the heterogeneous catalyst can be easily recycled up to six times while maintaining high catalytic activity. Interestingly, and providing a basis for mechanistic studies, the reaction does not proceed properly under N_2_ conditions or in the dark. Two reactions of the same type have been reported, with good results obtained with visible light-induced homogeneous catalytic systems using Rhodamine 6G and Rhodamine B as photocatalysts, although in non-recyclable reactions [45,46].

**Scheme 8:**
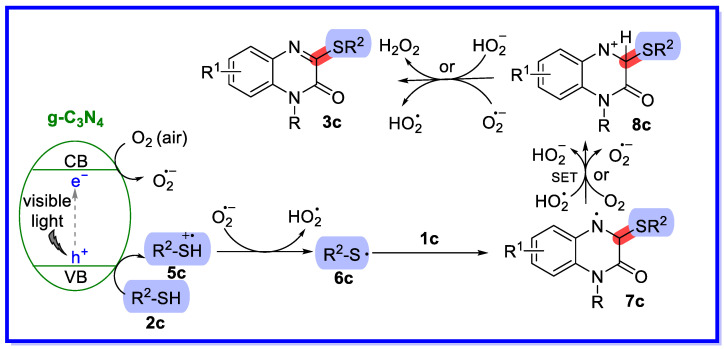
Reaction mechanism of the g-C_3_N_4_-catalyzed sulfonation of quinolin-2(1*H*)-ones.

A possible reaction mechanism has been proposed based on control experiments and related precedents in the literature (Scheme 8) [44]. First, g-C_3_N_4_ is excited by visible light irradiation, producing holes in the valence band (VB) and electrons in the conduction band (CB). The holes then acquire an electron from the thiol **2c** to form the thiyl radical cation **5c** via single electron transfer (SET). At the same time, the electrons in the CB are transferred to O_2_ (air) to form O_2_^•−^. Next, O_2_^•−^ abstracted hydrogen from the thiyl radical cation **5c** forms HO_2_• species and the thiyl radical **6c**, which adds to the C=N of **1a** to provide the nitrogen radical intermediate **7c**. Intermediate **7c** undergoes further oxidation by HO_2_• or O_2_ to provide the intermediate **8c**. Finally, product **3c** is obtained by deprotonation of the nitrogen cation intermediate **7c**.

**Scheme 9:**
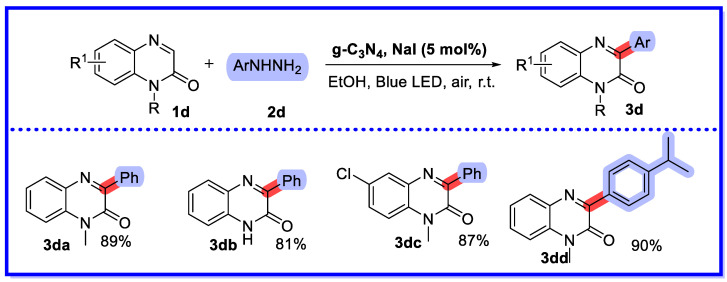
g-C_3_N_4_/NaI semi-heterogeneous bis-catalysed quinoxaline-2(1*H*)-ones and arylhydrazines.

In 2023, He et al. first proposed the formation of 3-arylquinoxaline-2(1*H*)-ones under blue light irradiation using g-C_3_N_4_/NaI semi-heterogeneous bis-catalysed quinoxaline-2(1*H*)-ones and arylhydrazines (Scheme 9) [47]. Their method yielded a wide variety of 3-arylquinoxalin-2 (1*H*)-one products in high yields with good functional group tolerance. Similar to the g-C_3_N_4_ photocatalyst described above, the desired product was not detected in the absence of molecular oxygen or light conditions. The reaction yield at the gram level was 72%. Cycling experiments with the g-C_3_N_4_ catalyst showed that it could be recovered and reused at least five times without significant loss of catalytic activity. In 2017, direct oxidative arylation homogeneous reactions of quinoxaline-2(1H)-ones prepared under air conditions without transition metal iodobenzene promotion were reported using different arylhydrazides as starting materials. In comparison, photocatalytic heterogeneous reactions are more environmentally friendly [48].

**Scheme 10:**
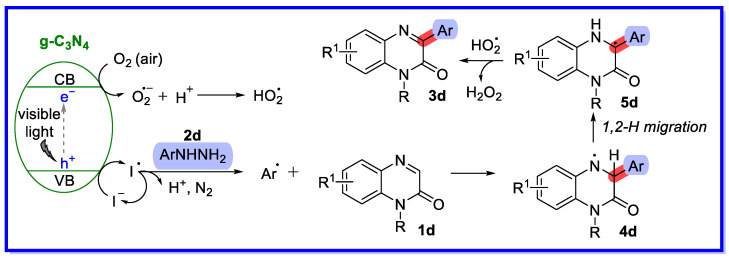
Reaction mechanism of g-C_3_N_4_/NaI semi-heterogeneous bicatalytic quinoxaline-2(1*H*)-ones.

A reaction mechanism similar to the one described above for g-C_3_N_4_ has been proposed by means of controlled experiments and has been reported in the literature (Scheme 10) [47]. First, blue light irradiation is used to induce charge separation in g-C_3_N_4_, producing a photogenerated hole (h^+^) and a photogenerated electron (e^−^). The h^+^ then oxidises the iodide ion to produce the iodine radical, which oxidises **2d** to produce the phenyl radical, molecular nitrogen, protons, and the same iodide ion. Simultaneously, molecular oxygen (in air) is reduced by the photogenerated electron to produce O_2_^•−^, which further combines with H^+^ to produce the hydroperoxide radical (HO_2_•). The phenyl radical region selectively attacks **1d** to form the N-centred radical **4d**, which undergoes 1,2-H migration to form the C-centred radical **4d**. Finally, the O_2_^•−^ abstracts a hydrogen atom from **5d** to provide the terminal product **3d** together with H_2_O_2_.

**Scheme 11:**
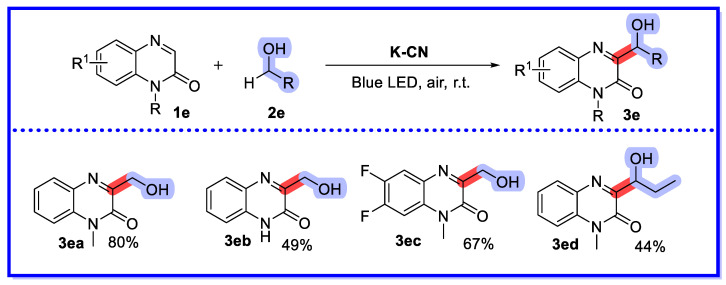
K-CN used as heterogeneous photocatalyst for hydroxyalkylation of quinoxalin-2(1*H*)-ones.

In 2022, Yu and his colleagues prepared potassium-modified carbon nitride (K-CN) directly from g-C_3_N_4_ at a lower calcination temperature and shorter calcination time, and used K-CN as a heterogeneous photocatalyst to achieve the first hydroxyalkylation of quinoxalin-2(1*H*)-ones under visible light irradiation using air as an external oxidant. (Scheme 11) [49] After investigating the catalytic performance, a molar ratio of KCl to LiCl of 0.56 and a calcination time of 1.5 h was determined to be the optimum catalyst, and the differences between the optimum catalyst and g-C_3_N_4_ were compared using a range of characterisation tools. In order to understand the significantly different catalytic activity observed for K-CN_0.56_-1.5 in this reaction, the authors performed a comparative study of K-CN_0.56_-1.5 and g-C_3_N_4_. The functional groups in K-CN_0.56_-1.5 were examined using FT-IR spectroscopy; two peaks located at 805 cm^−1^ and 2177 cm^−1^ were similar to the FT-IR spectra of g-C_3_N_4_, while two other peaks at 914 cm^−1^ and 993 cm^−1^ were associated with the addition of K^+^ and the presence of electron-absorbing nitrile groups. Notably, in the spectrum of K-CN_0.56_-1.5 the band belonging to N-H is weaker than that of g-C_3_N_4_, suggesting that the K^+^ is linked to the N atom. The XRD spectra of K-CN_0.56_-1 samples were similar to the other K-CN. The XPS results were in agreement with the FT-IR analysis, confirming that K^+^ was successfully doped. The morphology of K-CN_0.56_-1. was compared with that of g-C_3_N_4_ by SEM, with g-C_3_N_4_ showing an aggregated stacked lamellar structure and K-CN_0.56_-1 showing an aggregated granular lamellar structure. In addition, the transient photocurrent (TPC) response confirmed the high photocatalytic activity of K-CN_0.56_-1. Using optimal reaction conditions, a number of substrates were screened, providing moderate to excellent yields. It is worth noting that mono-alcohols can complete the reaction, whereas diols are not available as corresponding products. The cycling experiments showed a constant loss of potassium ions from the K-CN catalyst. With the addition of two equivalents of KF, a yield of 76% was obtained in the second recovery test; however, the yield of the desired product dropped significantly in the third run, probably due to the continuous loss of potassium ions.

**Scheme 12:**
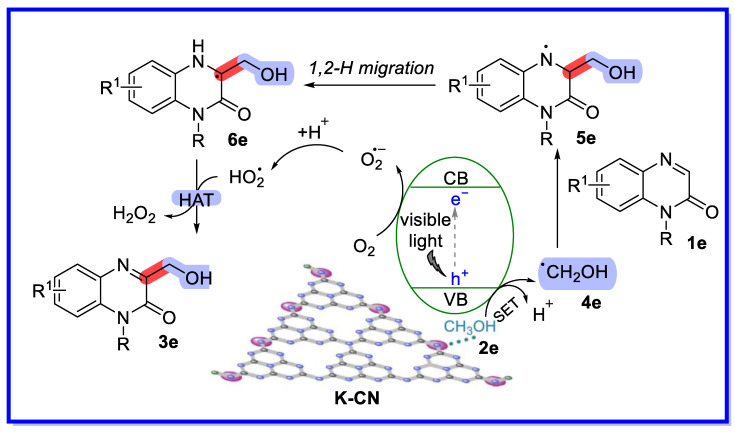
Mechanism of quinoxalin-2(1*H*)-one hydroxyl alkylation.

On the basis of the experimental results and the literature to date, a plausible mechanism is proposed in Scheme 12 [49]. First, the K-ion-activated MeOH reacts with the hole in the valence band (VB) of the excited photocatalyst to form the radical **4e**, with the loss of a proton through a single electron transfer (SET) process. Meanwhile, O_2_ is reduced by the electron in the conduction band (CB) to form O_2_^•−^, which combines with the proton to form HO_2_•. Subsequently, the radical **4e** is added to the C=N double bond of **1e** to provide the nitrogen-centred radical **5e**, which undergoes 1,2-H migration to access radical **6e**. Finally, the hydrogen atom transfer (HAT) process between radical **6e** and HO_2_• produces the target product **3e** and H_2_O_2_.

### 2.2. MOF/COF as Heterogeneous Catalysts

Metal-organic frameworks (MOFs) have turned up as intriguing series of crystalline materials, consisting of metal cations interconnected by multifunctionalised organic ligands during a self-assembly procedure [50,51,52]. Potential applications of these networks have expanded from gas capture and storage to other domains such as catalysis, conductivity, chemical sensors, optoelectronics, biomedical imaging, drug storage, and drug delivery [53,54].

After being first reported by Yaghi and colleagues in 2005, nine covalent organic frameworks (COFs) have received a great deal of attention in terms of synthesis, structure, fundamental properties, energy storage, and catalytic applications [55,56,57]. COFs have emerged as a new class of non-homogeneous photocatalysts for visible light-driven chemical conversions due to their high thermal and chemical stability, large surface area, and excellent optical properties. They have the inherent advantage of easy catalyst recovery over existing homogeneous photocatalysts, which is essential for practical applications in continuous chemistry [58,59,60].

**Scheme 13:**
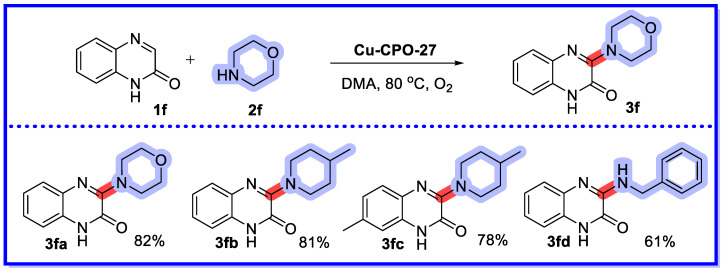
Cu-CPO-27 catalysis of the C–H amination of quinoxalin-2(1*H*)-ones.

In 2017, Phan’s group synthesised copper-based MOF Cu-CPO-27 for a recyclable heterogeneous catalytic reaction in which quinoxalin-2(1*H*)-ones undergo C–H amination with amines (Scheme 13) [61]. Alkylamines and benzylamines can be used as a source of amines, while aromatic amines do not react properly as a source of amines. A series of 3-aminoquinoxalin-2(1*H*)-ones were synthesised using molecular oxygen as the oxidant, and high yields were obtained. Fifteen substrates were reported with yields ranging from 42–89%. The authors compared the reactivity of Cu-CPO-27 with other MOFs for this reaction, and Cu-CPO-27 showed higher activity. At the end of the reaction there was no significant change in yield after five iterations of the reaction by washing and drying the catalyst. In addition, the recycled framework catalyst was characterised by FT-IR and XRD. The FT-IR spectrum of the recycled Cu-MOF showed an absorption band comparable to that of the new framework. The XRD analysis showed that the crystallinity of the catalyst remained constant during the experiment, although slight differences were detected in the repurposed framework. A copper-catalysed homogeneous reaction method for C-3 amination oxidation of quinoxalin-2(1*H*)-ones using primary or secondary amines as the nitrogen source has been reported as well. Compared with the above reactions, this synthetic strategy uses less catalyst, the heterogeneous catalysts are recyclable, and each has its own properties [62].

**Scheme 14:**
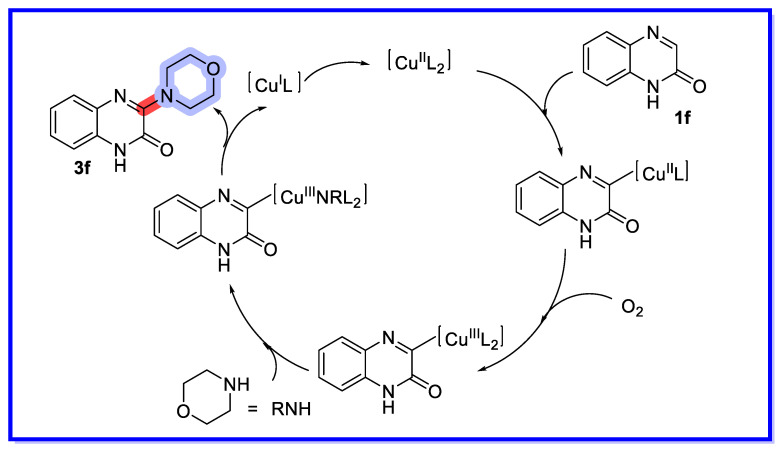
Proposed mechanism for the direct C–H amination reaction.

A possible reaction mechanism can be deduced from controlled experiments and literature reports (Scheme 14) [61]. Initially, deprotonation of quinoxalin-2(1*H*)-one (**1f**) forms the pyridinocopper(II) species. Subsequent oxidation of copper (II) by oxygen produces copper (III) species, which undergo nucleophilic substitution with morpholine to form aminocopper (III) species. Reductive elimination follows the single electron oxidation to regenerate the original catalyst. There is doubt as to the mechanism of this reaction. We think it unlikely that Cu^3+^ appears in the catalytic cycle, while it is not clear where the hydrogen disappears from in this scheme.

**Scheme 15:**
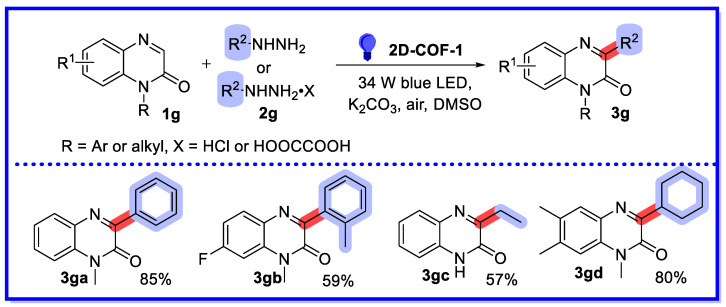
Visible light-driven arylation and alkylation of quinoxalin-2(1*H*)-ones.

In 2019, Yang’s group reported a visible light-driven arylation and alkylation reaction of quinoxalin-2(1*H*)-ones with hydrazines, using a hydrazine-based two-dimensional covalent organic framework (2D-COF-1) as a non-homogeneous photocatalyst (Scheme 15) [63]. Due to its excellent photocatalytic properties, good chemical stability, and heterogeneous nature, this method is characterised by high efficiency, good functional group tolerance, easy scalability, and reusable catalysts. Twenty-three substrates were reported, with yields ranging from 41–85%. Importantly, this provides an alternative method for rapid access to a variety of C3 arylated or alkylated quinoxalin-2(1*H*)-ones in a more environmentally friendly and sustainable manner. Note that the reaction cannot take place under an argon atmosphere, which means that oxygen in the air may play a key role in the current photocatalytic cycle. Photocatalyst recycling experiments showed that 2D-COF-1 maintained high photocatalytic activity after six runs.

**Scheme 16:**
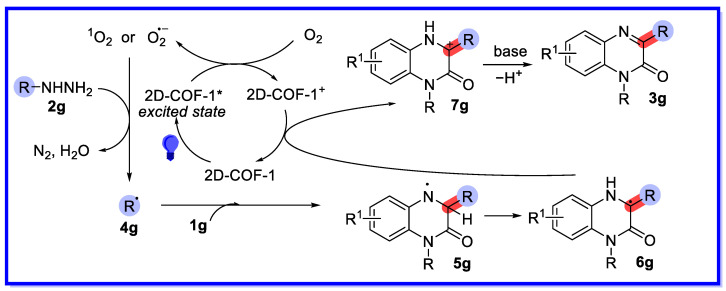
Mechanisms of arylation and alkylation of quinoxalin-2(1*H*)-ones driven by visible light.

A possible heterogeneous visible light-accelerated C3 arylation and alkylation reaction pathway has been suggested by radical inhibition experiments, calculation of apparent quantum efficiency (450 nm) values, and other relevant reports (Scheme 16) [63]. First, 2D-COF-1 is excited by the blue LED irradiation and subsequently reduces the oxygen to form a singlet oxygen radical and a superoxide radical anion, which oxidises the hydrazine **2g** with the loss of nitrogen and water to provide the nucleophilic cyclohexyl radical **4g**. Next, it reacts with the electron-deficient **1g** to provide the radical intermediate **5g**. The 1,2-H shift of radical **5g** then provides the carbon radical intermediate **6g**. Intermediate **6g** undergoes single electron transfer (SET) with 2D-COF-1^+^ to provide the carbon cation intermediate **7g**. This is finally converted by deprotonation to the desired product **3g**.

**Scheme 17:**
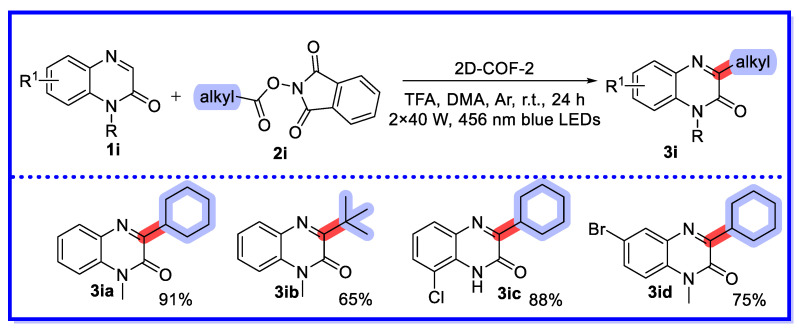
Visible light-driven decarboxylative alkylation reaction of heterocycles.

In 2021, Yang’s group described a visible light-driven decarboxylative alkylation reaction of heterocycles, including quinoxalin-2(1*H*)-one, catalysed by an olefin-linked covalent organic framework (2D-COF-2) instead of the commonly used noble metal complexes and organic dyes (Scheme 17) [64]. A variety of alkylated heterocycles were selectively and efficiently synthesised under non-homogeneous reaction conditions. Twenty-seven quinoxalin-2(1*H*)-one substrates were reported, with yields ranging from 61–97%. 2D-COF-2 relies on the super-stability of the olefin chains to maintain its basic structure and photoactivity under strongly acidic conditions. Furthermore, the structure, porosity, and crystallinity of the catalysts were confirmed by ^13^C solid-state NMR spectroscopy, Fourier transform infrared (FT-IR) spectroscopy, N_2_ adsorption measurements, and powder X-ray diffraction (PXRD). In addition, its potential for industrialisation in recycling experiments, functionalisation of bioactive molecules, and scale-up reactions has been demonstrated. Homogeneous photocatalysts for the same type of reaction have been limited to noble metal complexes and organic dyes, and the heterogeneous approach is advantageous from a green and sustainable chemistry perspective.

**Scheme 18:**
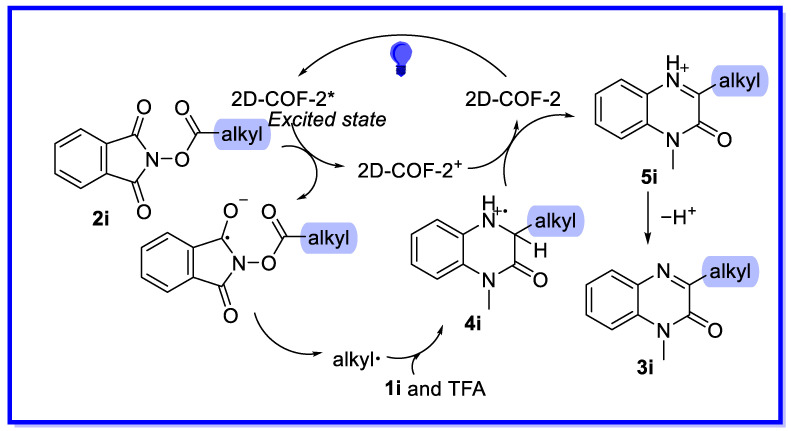
Mechanism of visible light-driven decarboxylative alkylation of heterocycles.

Combining controlled experiments and their inherent optoelectronic properties, a preliminary mechanism can be proposed (Scheme 18) [64]. Under visible light irradiation, the excited 2D-COF-2 can directly reduce the NHPI ester **2i** by single electron transfer to form alkyl radicals with loss of CO_2_. The alkyl radical then attacks the low electron density site on isoquinoline **1i**, which is activated by TFA, resulting in the formation of the radical cation intermediate **4i**. Intermediate **4i** is then oxidised by 2D-COF-2^+^ to provide the alkylated isoquinoline cation **5i** with photocatalyst regeneration. Finally, the desired product **3i** is obtained by deprotonation.

**Scheme 19:**
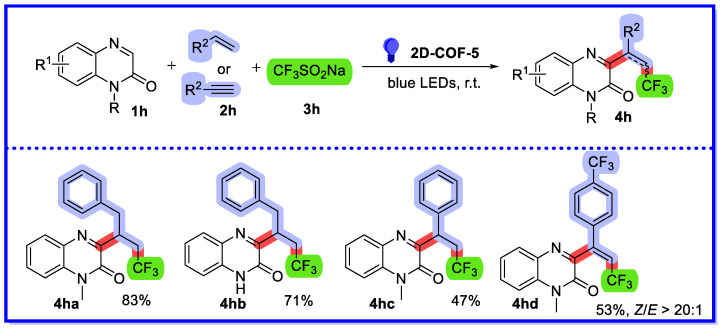
Visible light-driven heterogeneous catalytic three-component reaction of quinoxalin-2(1*H*)-one.

In 2022, Yang and colleagues reported a visible light-driven heterogeneous catalytic three-component cascade reaction of alkene or alkyne, CF_3_SO_2_Na, and quinoxalin-2(1*H*)-one (Scheme 19) [65]. In this reaction, two-dimensional imine-linked covalent organic frameworks (2D-COF-5) were used as heterogeneous photocatalysts. A wide range of C3 trifluoroalkyl and trifluoroalkenyl quinoxalin-2(1*H*)-one derivatives were prepared in satisfactory yields. Thirty-four substrates were reported, with yields ranging from 37–83%. The catalytic efficiency, basic structure, crystallinity, and microscopic appearance remained unchanged after eight cycles, although the BET surface area decreased. Particularly surprising was the high *Z*/*E* selectivity of the reaction of quinoxalin-2(1*H*)-one with alkynes due to the mild reaction conditions. The *Z*/*E* ratio was higher than 10:1, demonstrating good *Z* selectivity. The same type of reaction has been reported using 4CzIPN as a homogeneous photocatalyst and a cascade reaction initiated by visible light irradiation. Although considerable progress has been made in the synthesis of homogeneous reactions, there is much room for improvement in terms of sustainability [66].

**Scheme 20:**
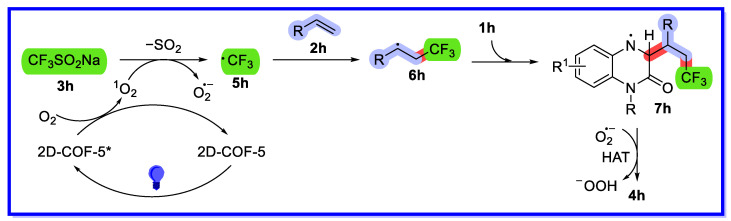
Three-component reaction mechanism of visible light-driven heterogeneous catalytic quinoxalin-2(1*H*)-one.

Based on controlled experiments and previous related studies, a plausible response mechanism for this heterogeneous visible light-induced three-component system is outlined in Scheme 20 [65]. First, 2D-COF-5* is excited by blue LED irradiation and activates oxygen in air to ^1^O_2_ via energy transfer (ET). Then, the singlet oxygen oxidises CF_3_SO_2_Na to produce the CF_3_ radical **5h**, with simultaneous loss of SO_2_. Simultaneously, O_2_^•−^ is produced via single electron transfer (SET). Subsequently, the CF_3_ radical is trapped by the olefin to produce the carbon radical intermediate **6h**. Intermediate **6h** then undergoes regioselective addition at the C3 position of quinoxalin-2(1*H*)-one **1h** to provide the free radical intermediate **7h**. Finally, hydrogen atom abstraction (HAT) occurs between intermediate **7h** and O_2_^•−^ to provide the desired product **4h**.

### 2.3. Ion-Exchange Resins as Heterogeneous Catalysts

Ion exchange resin catalysts have gained much attention because of their low cost, metal-free composition, easy product purification, recyclability, and reusability [67,68,69].

**Scheme 21:**
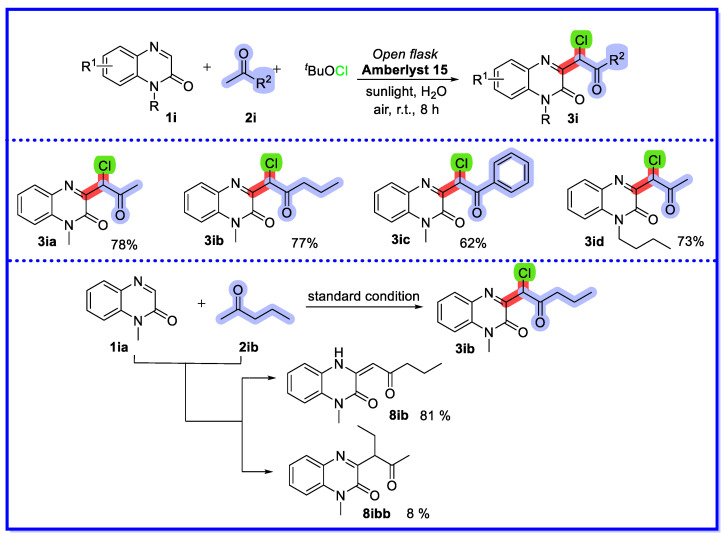
Sunlight-induced heterogeneous catalytic multicomponent reactions of quinoxalin-2(1*H*)-ones.

In 2021, Zhang’s group demonstrated a non-homogeneous synthetic method for sunlight-catalysed multicomponent transformations in water using quinoxalin-2(1*H*)-one as a reactant for the bifunctionalisation of methyl ketones (Scheme 21) [70]. In this transformation, the ion exchange resin Amberlyst 15 was used as a multiphase catalyst due to its recyclability and reusability. This method provides a green and sustainable route for the synthesis of various alpha-bifunctionalised ketones in medium to high yields. It should be noted that when using an unsymmetrical aliphatic ketone such as 2-pentanone as the starting material, a small amount of product **8ibb** is formed. The authors suggest two possible reasons for this result. On the one hand, although more substituents favour enolisation, the greater the site resistance is not favourable for the reaction. On the other hand, compounds **8ib** and **8ibb** are competitive, and compound **8ib** can be further converted to the final product **3ib**. Thus, according to the Le Chatelier’s principle, the formation of compound **8ib** is more favourable, making compound **3ib** the main product. In addition, the higher yields of the aromatic ketone conversion products with the addition of Triton X-100 as surfactant may be due to the increased water solubility of the aromatic ketones by the surfactant. After completion of the conversion, the mixture was extracted directly with ethyl acetate, while the aqueous phase containing the catalyst was used to facilitate the reaction by direct addition of the starting material. Cycling experiments showed that a significant loss of catalytic activity was observed in the third cycle. However, reprocessing the catalytic system (which had been reused four times) with a simple washing method allowed the catalyst to be reactivated with better catalytic activity.

**Scheme 22:**
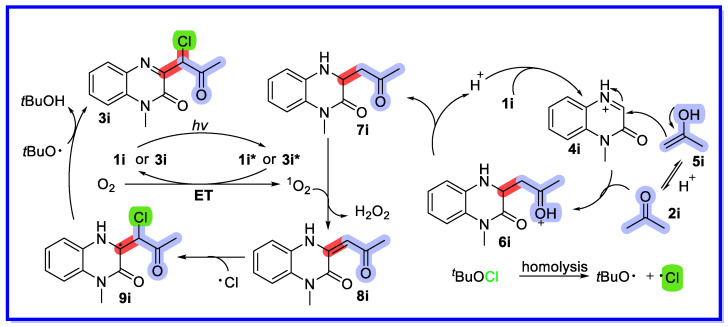
Mechanism of sunlight-induced heterogeneous catalytic multicomponent reactions of quinoxalin-2(1*H*)-ones.

Based on previous reports and the results of control experiments, a reasonable mechanism has been proposed (Scheme 22) [70]. First, protonation of substrate **1a** with protonic acid provides an iminium ion **4i**, which reacts with the enol form **5i** to provide the intermediate **6i**. Intermediate **6i** is then converted by deprotonation to the Mannich-type product **7i**. Meanwhile, the starting material **1i** (or **3i**) is excited by sunlight to form the excited species 1i* (or 3i*), which acts as a photocatalyst to undergo an energy transfer (ET) process with triplet oxygen (^3^O_2_) to provide singlet oxygen (^1^O_2_) together with reformation of the ground state **1i**. The Mannich-type product **7i** is oxidised to compound **8i** by an oxidative dehydrogenation process enabled by singlet oxygen (^1^O_2_). The chlorine radical (Cl•) generated by the homolysis of *^t^*BuOCl then attacks compound **8i** to provide intermediate **9i**. The final product **3i** is obtained by oxidation of the intermediate **9i** in the presence of tert-butoxy (*^t^*BuO•). The corresponding product can act as a photosensitiser to produce singlet oxygen (^1^O_2_).

**Scheme 23:**
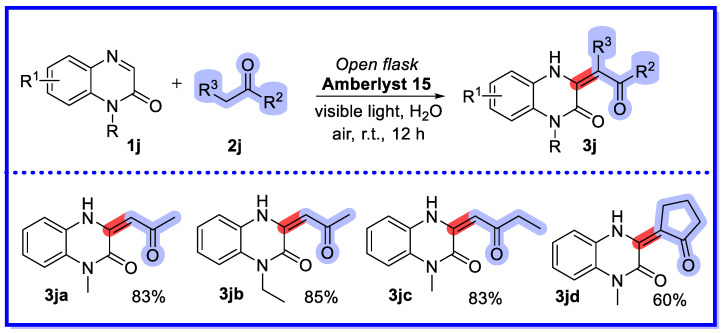
Enolization of quinoxalin-2(1*H*)-ones in water.

In the same year, the Zhang group reported a reusable sulfonic ion exchange resin (Amberlyst 15)-facilitated photocatalytic approach for the direct enolization of quinoxalin-2(1*H*)-ones in water (Scheme 23) [71]. The recoverable aqueous phase reaction was carried out under milder conditions, providing a green and practical route for the production of various (*Z*)-amino ketones containing a 3,4-dihydroquinoxalin-2(1*H*)-one skeleton. This method successfully combines the heterogeneous Mannich reaction with photocatalysis. At the end of the reaction, the organic layer containing the product was purified by extracting the reaction mixture with ethyl acetate. The starting material was then added directly and the aqueous phase was used to catalyse the conversion. A slight loss of catalytic activity was observed in the second run, and a significant loss of catalytic activity was observed in the third and fourth runs. Reprocessing the catalysts after three or four runs regenerated the catalysts with better catalytic activity.

**Scheme 24:**
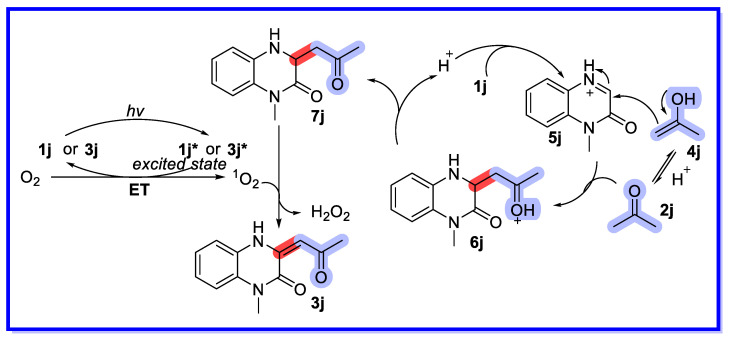
Mechanism of enolization of quinoxalin-2(1*H*)-ones in water.

Based on mechanistic studies and previous reports, a plausible mechanism for this reaction is proposed in Scheme 24 [71]. In this conversion, Amberlyst 15 acts as a protonic acid to catalyse a Mannich-type reaction. First, acetone **2j** is converted to the enol form **4j**, which is then attacked by the protonated imine **5j** to provide the intermediate **6j**. The Mannich-type product **7j** is then formed by deprotonation of intermediate **6j**. Finally, the reaction undergoes two SET processes with the singlet oxygen ^1^O_2_ to provide the target product together with H_2_O_2_ (detected by hydrogen peroxide test paper). Intermediate **7j** and the resulting product **3j** can be used as photocatalysts to facilitate the photocatalytic cycle.

**Scheme 25:**
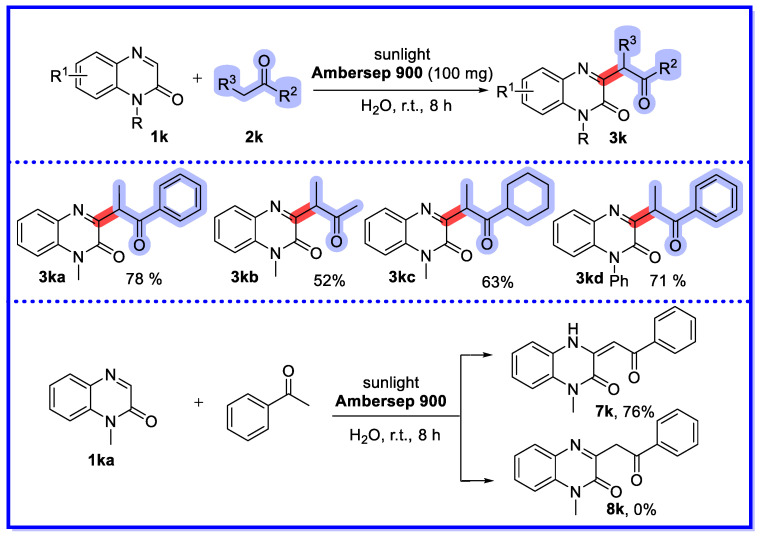
Ambersep 900-catalysed strategy for α-heteroarylation of ketones with quinoxalin-2(1*H*)-ones.

In 2022, Li’s group (Scheme 25) [72] published a similar reaction method to that of Zhang [71], that is, a sunlight-promoted recyclable ion exchange resin (Ambersep 900)-catalysed strategy for the α-heteroarylation of ketones with quinoxalin-2(1*H*)-ones in the aqueous phase. In particular, good regioselectivity was observed in this reaction, suggesting that the ketone of the secondary carbon was the active site. The method was well adapted to the substrate, and a variety of α-heteroarylated products of ketones were synthesised. The recoverability of the catalytic system of the method were essentially similar to those previously reported by Zhang [71]. The authors used the reaction of methyl ketone and quinoxalin-2(1*H*)-ones, and the product **7k** was isolated with 76% yield, while the target product **8k** was not detected. It may have been the case that the energy required to form compound **7k** was less than that required for compound **8k**.

**Scheme 26:**
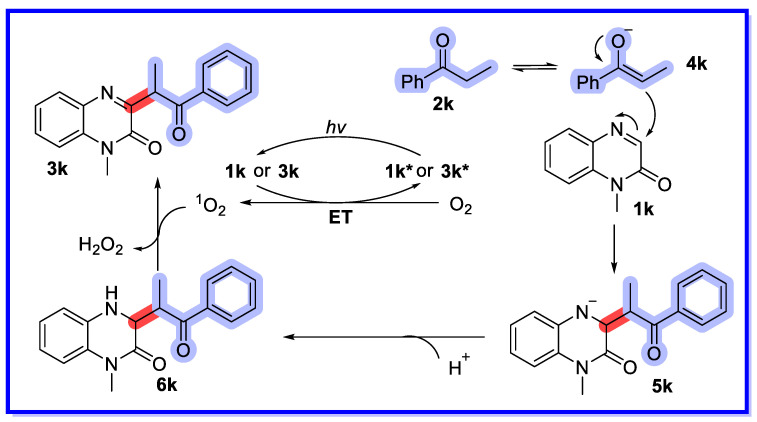
Mechanism of Ambersep 900-catalysed strategy.

Based on previous reports and mechanism studies, the same authors proposed a plausible mechanism for the α-functionalisation of ketones (Scheme 26). First, the starting material **2k** is converted to the enol form **4k** under alkaline conditions, then substrate **2k** reacts with **4k** in a Mannich-type reaction to provide the intermediate **5k**. Then, protonation of **5k** with H_2_O provides the Mannich product **6k**. Meanwhile, compound **1k** or **3k** is activated by sunlight to provide the excited species 1k* or 3k*, which can act as a photocatalyst to produce singlet oxygen from triplet oxygen via energy transfer (ET). Finally, the reaction proceeds through an SET process with singlet oxygen to produce the target product **3k** and generate H_2_O_2_, which can be detected using H_2_O_2_ test paper.

The selectivity of the reaction of quinoxalin-2(1*H*)-one, another asymmetric aliphatic ketone, with ion-exchange resins is highly dependent on the type of ion exchange resin as well as on the stability of the enolisation and the site resistance. Amberlyst 15 reacts readily with ketones with few substituents at the α-position to form **3j**, whereas Ambersep 900 reacts readily with ketones with many substituents at the α-position to form **3k**.

### 2.4. Other Heterogeneous Catalysts

**Scheme 27:**
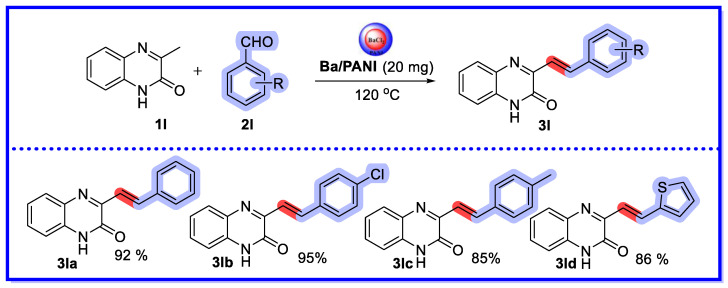
Ba/PANI nanocomposites as heterogeneous mesoporous nanocatalysts.

In 2022, Kapoor et al. used barium chloride-embedded polyaniline (Ba/PANI) nanocomposites as heterogeneous mesoporous nanocatalysts for the preparation of bioactive (*E*)-3-substituted styrylquinoxalin-2(1*H*)-ones from 3-methylquinoxalin-2(1*H*)-one and substituted aldehydes under solvent-free conditions (Scheme 27) [73]. The results showed that the catalytic activity of the Ba/PANI nanocatalysts was significantly higher than that of BaCl_2_ and PANI; moreover, the catalytic activity was easily recoverable, and remained high after five repeated uses. The TON for the template substrate was 28.68 and the TOF was 191.2 h^−1^. The authors characterized the synthesized barium-containing nanocomposites using XRD, FTIR, SEM, HR-TEM, EDX, ICP-MS, UV-Vis, TGA, N_2_ adsorption-desorption isotherm (BET), and XPS. TEM analysis confirmed that the average grain size of the barium chloride nanoparticles in the composites was 13–14 nm. BET analysis confirmed that the mesoporous structure of the Ba/PANI nanocomposites played an important role in their catalytic efficiency. XRD and HRTEM analyses showed that the nanocomposites were polycrystalline in nature. ICP-MS analysis confirmed that 22.51 wt% of Ba was loaded on PANI. This method has the advantages of high yield, wide range of substrates, short reaction time, easy preparation, and simple handling of the catalysts.

**Scheme 28:**
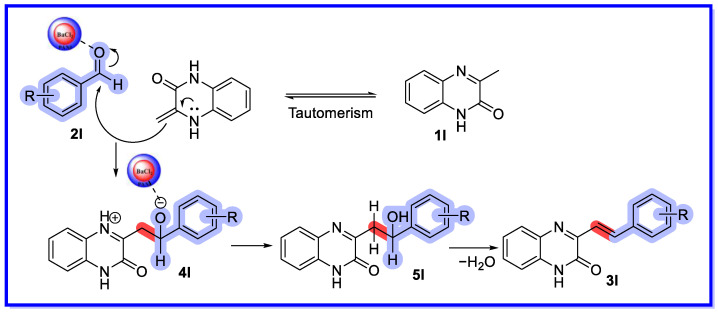
Mechanism of Ba/PANI nanocomposites as heterogeneous mesoporous nanocatalysts.

A mechanism for the synthesis of 3-substituted styrylquinoxalin-2(1*H*)-ones can be proposed based on reports in the literature (Scheme 28) [73]. Ba/PANI acts as a Lewis acid and activates the carbonyl group of the aldehyde (**2l**), thereby facilitating the nucleophilic attack of 3-methylquinoxalin-2(1*H*)-one (**1l**) through the intermediate **4l** to provide the intermediate **5l**. Finally, **5l** is dehydrated to provide the desired product **3l**.

**Scheme 29:**
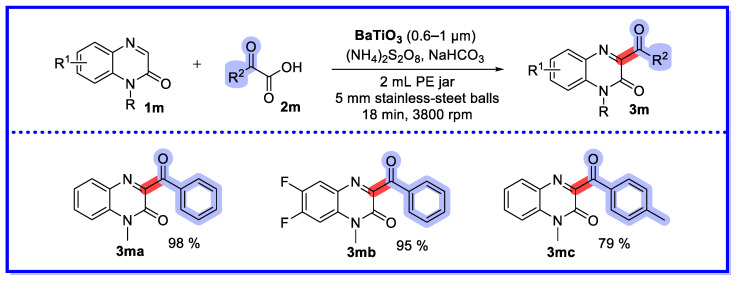
Piezochemically-driven decarboxylation coupling of C–H bonds.

In 2023, Yuan’s group developed the first piezochemically-driven decarboxylation coupling of C–H bonds (Scheme 29) [74]. Stirring BaTiO_3_ by ball milling converts mechanical energy into electrical potential, leading to the production of benzoyl radicals via a single electron transfer pathway similar to photocatalytic reactions. This mechanical redox BaTiO_3_-catalysed synthesis of C3 acylated quinoxalin-2(1*H*)-ones using α-oxocarboxylic acids as acylation reagents is a solvent-free strategy with short reaction times and simple operation, and has promising applications in large-scale chemical production. In addition, BaTiO_3_ can be reused three times under optimal reaction conditions without significant loss of product yield. To further investigate the piezoelectric effect, the authors carried out SEM and XRD analyses of BaTiO_3_ samples from the first, second, third, and fourth cycles. SEM showed that after the first cycle BaTiO_3_ grains began to break down into smaller sizes with irregularities on the grain surface. The size of the secondary grains continued to decrease. After the third cycle, the size of these loose soft agglomerates increased significantly. XRD showed that the diffraction peak of BaTiO_3_ decreased with an increasing number of cycles, while the lattice parameters did not change significantly. This was in agreement with the SEM results. Hu and colleagues reported a reaction of the same type involving the silver-catalysed decarboxylation of α-oxocarboxylic acids with quinoxalin-2(1*H*)-ones to provide 3-acylquinoxalin-2(1*H*)-one derivatives. Compared to this reaction, the ball milling reaction is more environmentally friendly, as it is solvent-free [75].

**Scheme 30:**
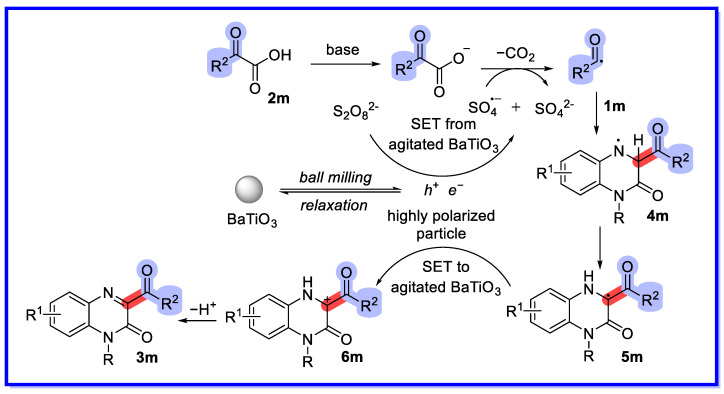
Mechanism of piezochemically-driven decarboxylation coupling of C–H bonds.

A plausible mechanism can be proposed, shown in Scheme 30 [74]. Initially, the mechanical force introduced by ball milling agitates the piezoelectric material BaTiO_3_, creating a transient electrochemical potential. The highly polarised BaTiO_3_ reduces (NH_4_)_2_S_2_O_8_ via single electron transfer (SET), leading to the formation of a free sulphate radical (SO_4_^•−^). The free sulphate radical is an even more aggressive oxidant than the persulphate anion. The presence of a base can accelerate the decomposition of persulphate into sulphate free radicals. The strong oxidant SO_4_^•−^ then accepts a single electron from the 2-oxo-2-phenylacetic acid anion, resulting in the formation of a sulphate anion (SO_4_^2−^) and corresponding benzoyl radical, with extrusion of CO_2_ as a byproduct. Addition of the benzoyl radical to quinoxalin-2(1*H*)-one **1m** leads to the nitrogen radical intermediate **4m**, which undergoes a 1,2-hydrogen shift to provide the carbon radical intermediate **5m**. Further oxidation of **5m** by holes present in the stirred piezocatalyst BaTiO_3_ yields the carbon cation intermediate **6m**. Finally, deprotonation of **6m** yields the desired product **3m**.

**Scheme 31:**
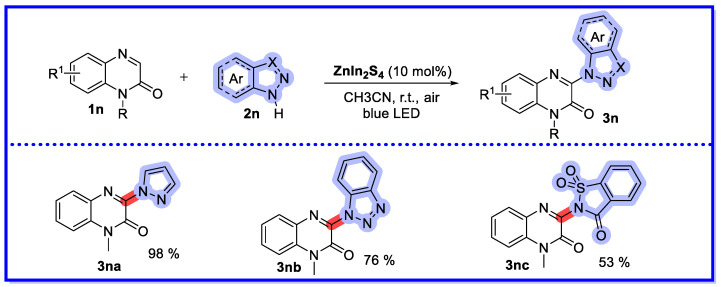
The direct C–H azoation of N-heterocyclic compounds.

In 2022, Yu and Chen’s group synthesised a series of indium zinc sulphide (ZnIn_2_S_4_) in different solvents using a solvothermal method (Scheme 31) [76]. ZnIn_2_S_4_ prepared in ethanol (ZIS-1) was characterised as having the highest photocurrent density. The direct C–H azoation of N-heterocyclic compounds (including quinoxalin-2(1*H*)-ones, quinazoline, and 2*H*-benzo[b]-[1,4]oxazin-2-ones) was achieved in an air atmosphere under 456 nm blue light, using ZIS-1 as a photocatalyst. More importantly, the ZIS-1 microsphere photocatalyst was stable during the catalytic process and could be used at least five times with no change in reactivity. The authors used PXRD to characterise the physical phases and crystal structures of the three synthesised ZnIn_2_S_4_ samples. The PXRD spectra of the three samples were almost identical, which is in agreement with previous reports. The UV-vis diffuse reflectance spectra of the synthesised ZnIn_2_S_4_ showed a significant broadening of the absorption band in the range of 400–500 nm, which favours light absorption in the visible region. The three catalysts consist of flower-like microspheres of about 4–5 μm in size. The microspheres consist of many interwoven nanosheets, and it can be seen that ZIS-1 appears to be somewhat looser than the other two catalysts. Further characterisation of the recovered ZIS-1 photocatalyst showed that the peaks and intensities of the recovered ZIS-1 remained almost unchanged compared to the newly synthesised ZIS-1. In addition, the recovered SEM showed that the surface morphology of the catalyst remained unchanged during the catalytic process, indicating that the photocatalyst ZIS-1 was stable during the catalytic process.

**Scheme 32:**
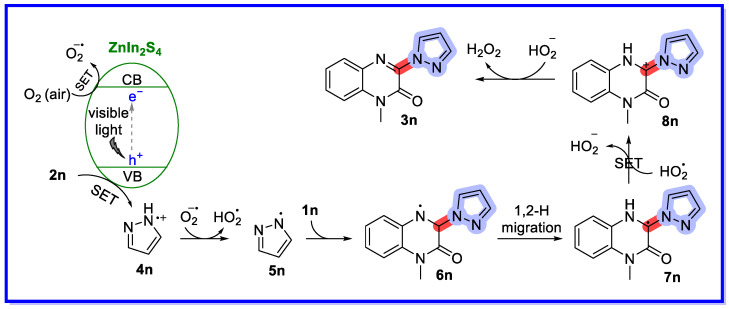
Mechanism of C–H azoation of N-heterocyclic compounds.

Based on the experimental results, the reaction mechanism in Scheme 32 was proposed [76]. First, ZIS-1 absorbs photons and produces electrons in the conduction band (CB) and causes holes in the valence band (VB). The positive oxidation potential makes it suitable for reaction with pyrazole (**2n**) to produce an N-centred pyrazol-1-yl radical cation **4n** via single electron transfer (SET). At the same time, the electrons in CB are transferred to O_2_ (air) by SET, producing a superoxide radical (O_2_^•−^). Then, **4n** is deprotonated by O_2_^•−^ to form the hydrogen peroxide radical (HO_2_•) and the N-radical intermediate **5n**. The N-radical intermediate **5n** is then incorporated into the C=N bond of quinoxalin-2(1*H*)-one **1n** to provide the intermediate **6n**, followed by 1,2-H migration to provide the carbon-centred radical intermediate **7n**. Next, **7n** is oxidised by HO_2_ via the SET pathway to produce the radical cation intermediate **8n**. Finally, the cation **8n** undergoes deprotonation to produce the desired product **3n**, while hydrogen peroxide (H_2_O_2_) is formed and can be detected by KI.

## 3. Summary and Outlook

The direct C–H functionalisation of quinoxalin-2(1*H*)-ones via heterogeneous catalytic reactions is a green and sustainable chemical reaction, a simple and effective approach that has attracted increasing interest from synthetic chemists and holds great promise for medicinal chemistry and functional materials. This paper reviews the heterogeneous catalytic reactions reported in recent years for the C–H functionalisation of quinoxalin-2(1*H*)-ones via different heterogeneous catalysts (e.g., graphitic phase carbon nitride, MOF, COF, ion exchange resins, piezoelectric materials, and microspheres) and shows the mechanisms and representative substrates of the mainly radical-based reactions in order to provide a more in-depth and rapid understanding of these reactions. At the same time, it can provide a reference for the further development of heterogeneous catalytic reactions of quinoxalin-2(1*H*)-ones.

Despite these outstanding achievements, relatively few heterogeneous catalytic reactions of quinoxalin-2(1*H*)-ones have been reported, and further development of heterogeneous catalytic reactions of quinoxalin-2(1*H*)-ones is essential. First, the types of heterogeneous catalytic reactions of quinoxalin-2(1*H*)-ones are very homogeneous, mainly involving the formation of C-C, C-N, C-S, and C-O bonds. In particular, the C-B/SI/P/RF/X/SE bond at the C3 position of quinoxalin-2(1*H*)-ones has not been completed, and is expected to be completed by the heterogeneous catalytic reaction of quinoxalin-2(1*H*)-ones. Second, the heterogeneous catalytic reactions of quinoxalin-2(1*H*)-ones use relatively few types of heterogeneous catalysts, such as metal oxides; graphene-based composites, doped metal nanoparticles, molecular sieve-based porous materials, single-atom catalysts, etc., have not yet been involved, and the types of heterogeneous catalysts that have been used to enantiosequence the reactions, e.g., piezoelectric materials, are very homogeneous as well and have only completed the acylation reactions. Third, asymmetric synthesis has been a research topic in organic equations; it is challenging to complete asymmetric catalytic reactions with quinoxalin-2(1*H*)-ones as substrates, and the introduction of chiral centres via inhomogeneous catalytic reactions of quinoxalin-2(1*H*)-ones is a concise route. Fourth, the importance of green chemistry metrics such as TOF and TON based on heterogeneous catalysts requires further elaboration. In summary, we hope that the review provided in this paper will be useful for research in this area. Heterogeneous catalytic reactions of quinoxalin-2(1*H*)-ones provide an atomically economical, environmentally friendly, and sustainable approach for the preparation of a wide range of functionalized quinoxalin-2(1*H*)-ones, with promising applications in materials science and medicinal chemistry.

## Data Availability

Not applicable.
